# Supplementary material for: Dominant and Priming Role of Waterlogging in Tomato at e[CO2] by Multivariate Analysis
Source: Int J Mol Sci. 2022 Oct 11;23(20):12121. doi: 10.3390/ijms232012121 (PMC9602540; doi:10.3390/ijms232012121)
Supplement: Supplementary file 1 [file ijms-23-12121-s001.zip › ijms-1789774-supplementary.pdf]

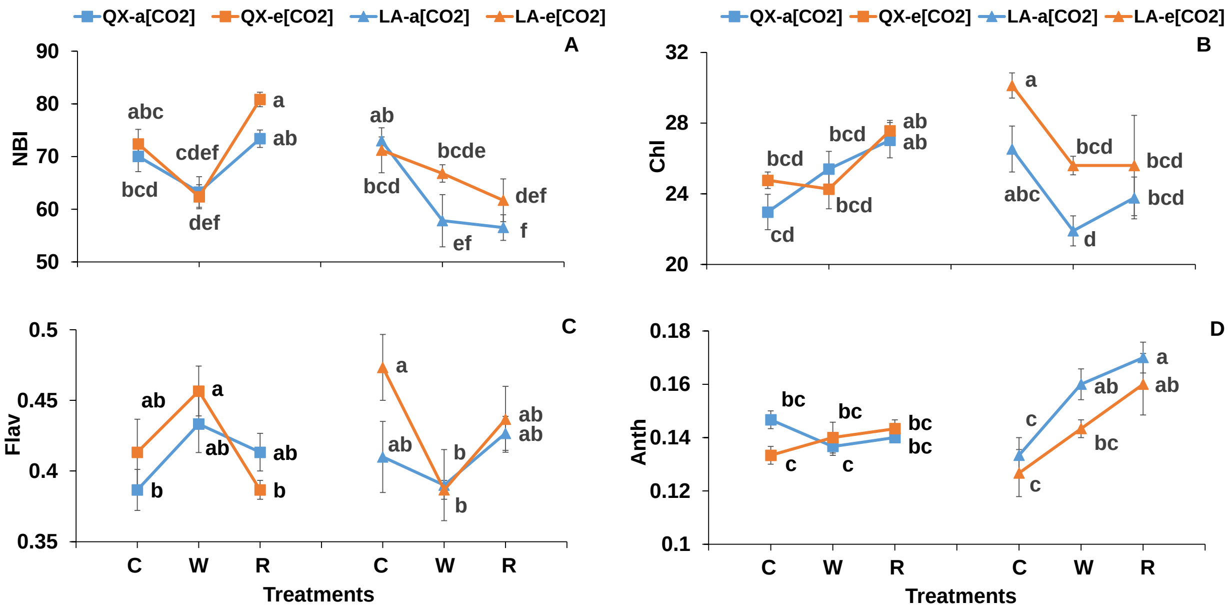

Supplementary Figure S1. (**A**) Nitrogen balanced index (NBI), (**B**) chlorophyll content (Chl), (**C**) flavonol content (Flav) and (**D**) anthocyanin content (Anth) of the first fully expanded leaf from the two tomato genotypes ('QX' and 'LA') after the first-round treatments for 4 days using Dualex (FORCE-A, Centre Universitaire Paris Sud, Cedex, France). The treatments, data analysis and presentation are the same as fig 1.

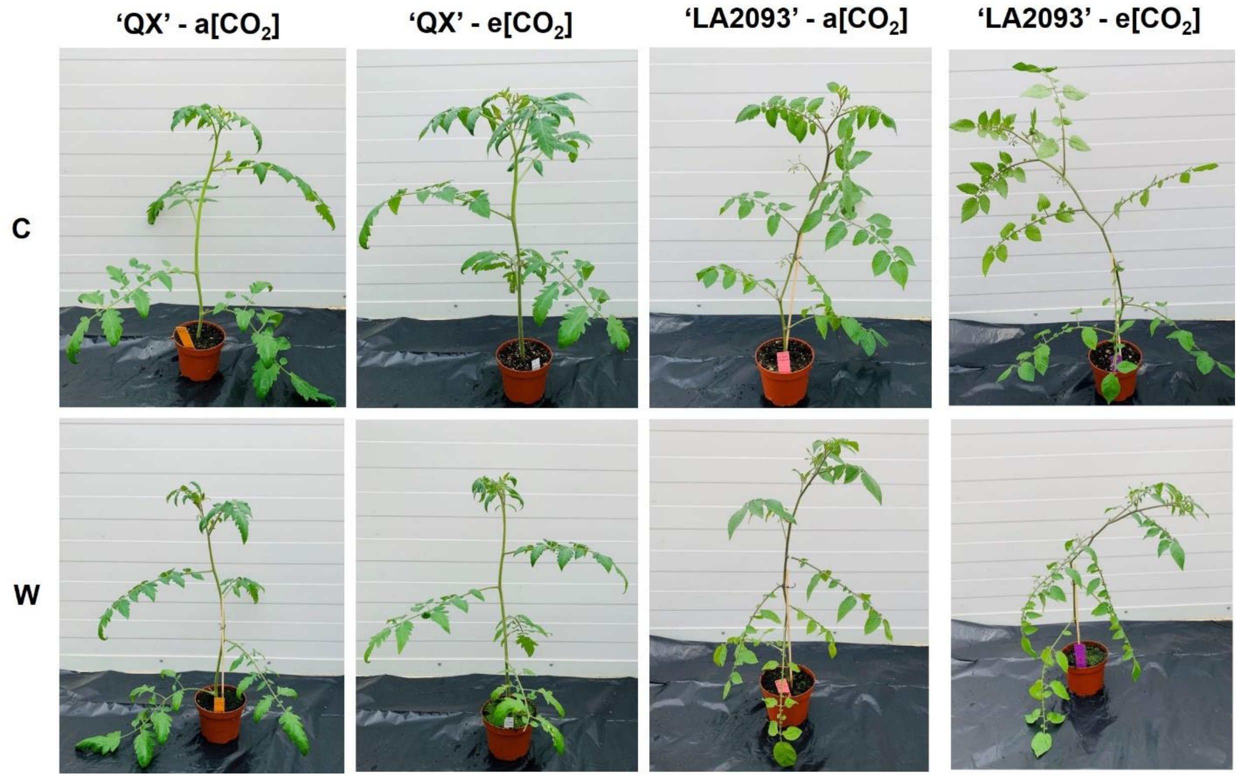

Supplementary Figure S2. The plant photos of the two tomato genotypes ('QX' and 'LA2093') after the first-round treatments for five days. The

$a[CO_2]$ and $e[CO_2]$ indicated 400 and 800 ppm CO_2 concentration, respectively. The ‘C’ and ‘W’ on the left side indicated that the plants were at control and waterlogging conditions, respectively. All the ANOVA was performed within all the treatments of two cultivars.

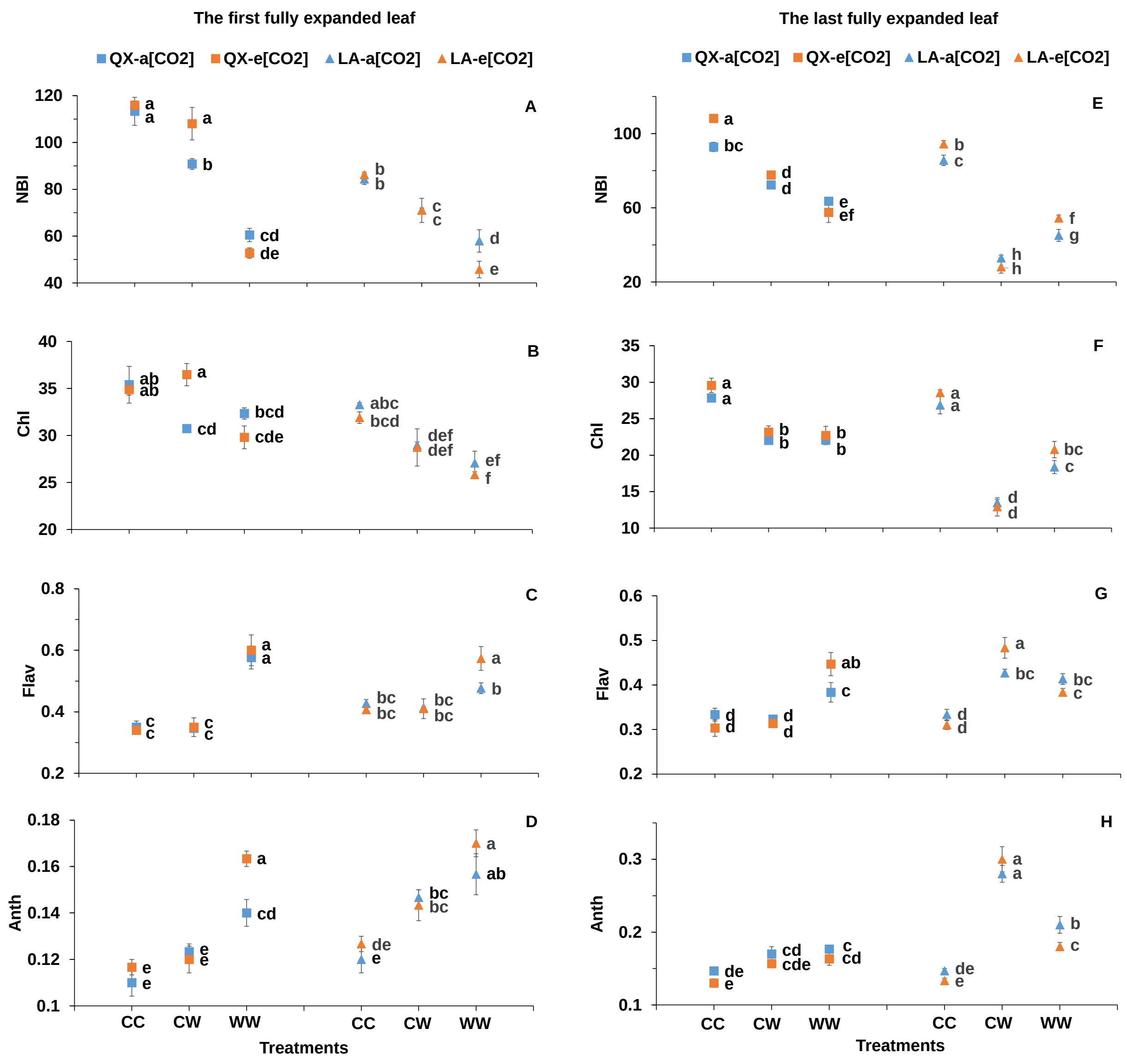

Supplementary Figure S3. Nitrogen balanced index (NBI), chlorophyll content (Chl), flavonol content (Flav) and anthocyanin content (Anth) of (**A**, **B**, **C**, **D**) the first and (**E**, **F**, **G**, **H**) the last fully expanded leaf from the two tomato genotypes ('QX' and 'LA2093') after the second-round treatments for four days using Dualex (FORCE-A, Centre Universitaire Paris Sud, Cedex, France). The treatments, data analysis and presentation are the same as fig 3.

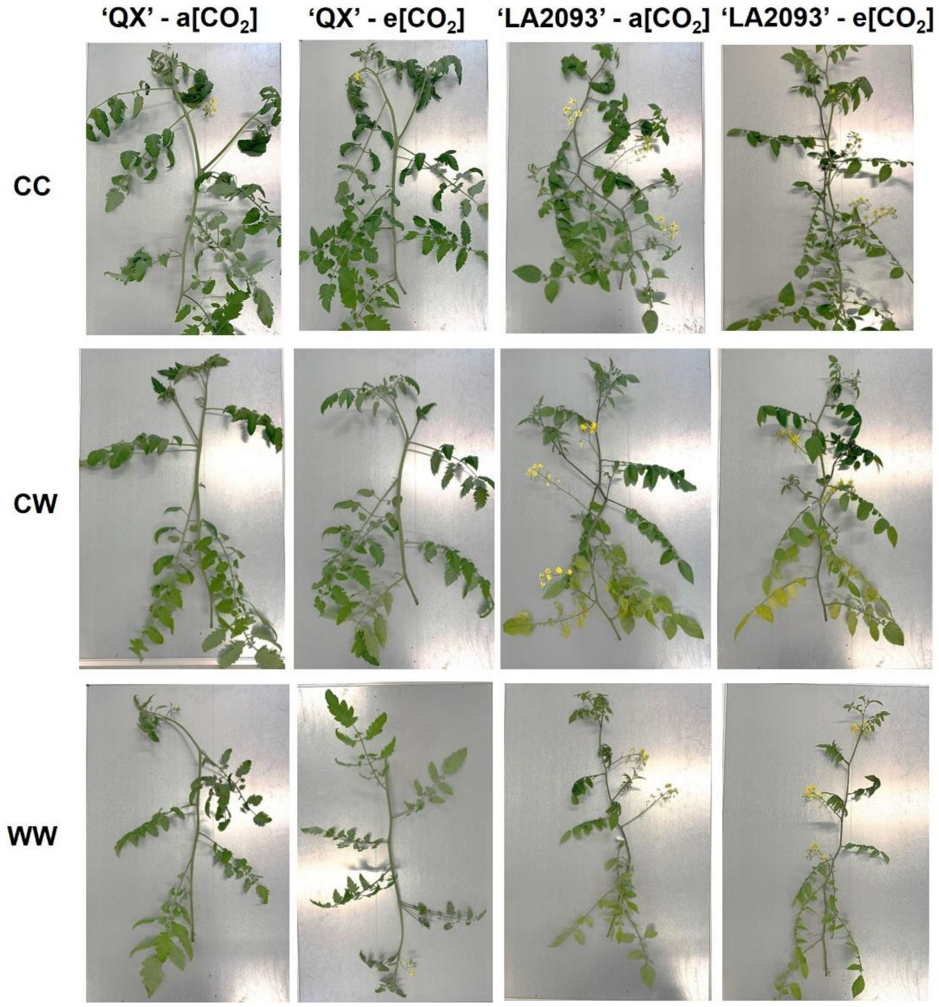

Supplementary Figure S4. The plant photos of the two tomato genotypes ('QX' and 'LA2093') after the second-round treatments for five days. The a[CO_2] and e[CO_2] indicated 400 and 800 ppm CO_2 concentration, respectively. The 'CC', 'CW' and 'WW' on the left side indicated that the plants were at (1) control condition for both rounds; (2) control followed by waterlogging condition; and (3) waterlogging condition for both

rounds, respectively. All the ANOVA was performed within all the treatments of two cultivars.

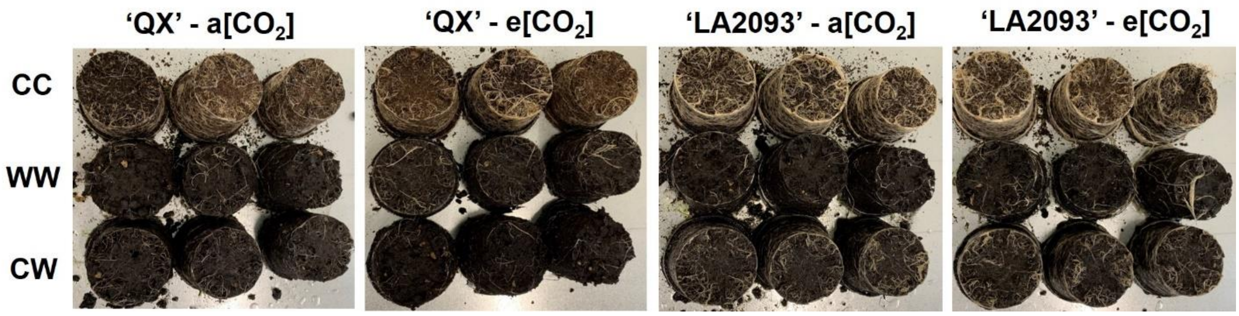

Supplementary Figure S5. The root photos of the two tomato genotypes ('QX' and 'LA2093') after the second-round treatments for five days. The a[CO_2] and e[CO_2] indicated 400 and 800 ppm CO_2 concentration, respectively. The 'CC', 'CW' and 'WW' on the left side indicated that the plants were at (1) control condition for both rounds; (2) control followed by waterlogging condition; and (3) waterlogging condition for both rounds, respectively.

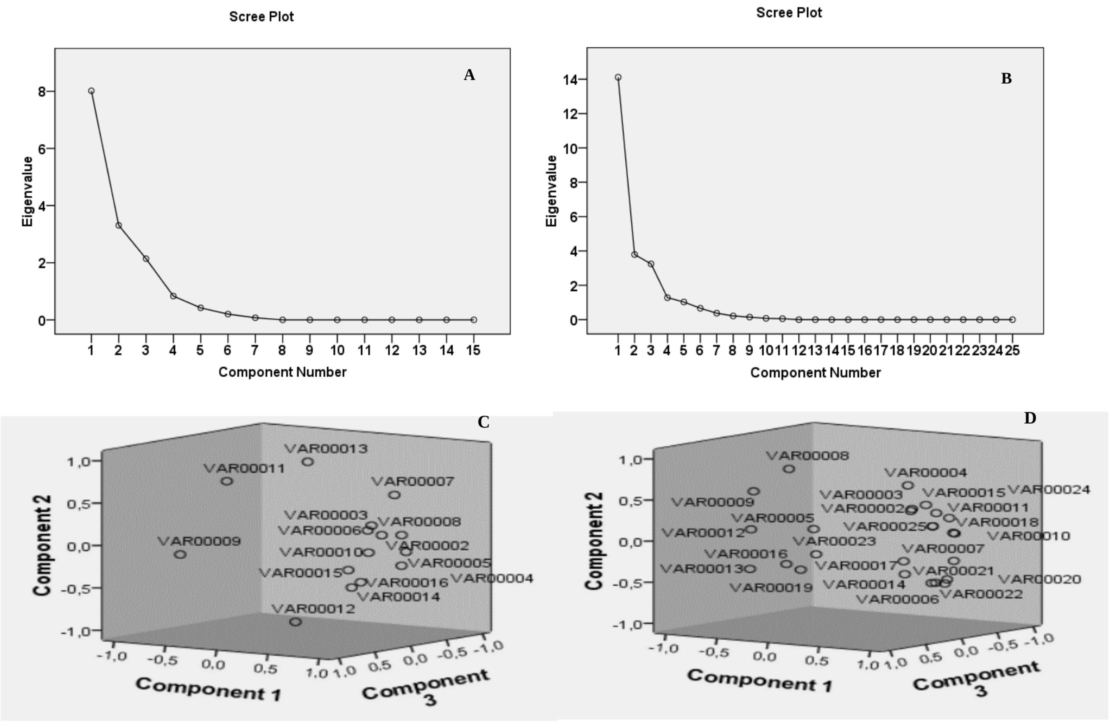

Supplementary Figure S6. Scree plot and principal component analysis (PCA) result based on (**A, C**) the first-round and (**B, D**) the second-round treatments
